# Refeeding Syndrome: A Critical Reality in Patients with Chronic Disease

**DOI:** 10.3390/nu14142859

**Published:** 2022-07-12

**Authors:** Gabija Krutkyte, Leyla Wenk, Jonas Odermatt, Philipp Schuetz, Zeno Stanga, Natalie Friedli

**Affiliations:** 1 Division of Diabetes, Endocrinology, Nutritional Medicine and Metabolism, Inselspital, Bern University Hospital, University of Bern, 3010 Bern, Switzerland; gabija.krutkyte@extern.insel.ch (G.K.); jonas.odermatt@insel.ch (J.O.); zeno.stanga@insel.ch (Z.S.); 2 Department of Anaesthesiology and Pain Medicine, Inselspital, University Hospital Bern, University of Bern, 3010 Bern, Switzerland; 3 Division of Paediatric Gastroenterology, Hepatology and Nutrition, Department of Paediatrics, Inselspital, Bern University Hospital, 3010 Bern, Switzerland; 4 Division of General Internal and Emergency Medicine, Medical University Department, Kantonsspital Aarau, 5001 Aarau, and Medical Faculty, University of Basel, 4056 Basel, Switzerland; philipp.schuetz@ksa.ch; 5 Department of Clinical Research, University Hospital Basel, University of Basel, 4056 Basel, Switzerland; 6 Medical Department, Hospital of Thun, 3600 Thun, Switzerland; natalie.friedli@spitalstsag.ch

**Keywords:** malnutrition, refeeding syndrome, nutritional support, catabolism

## Abstract

Malnutrition is one of the most frequent metabolic challenges in the population of chronically ill patients. This results in increased administration of nutritional therapy in inpatient settings, which poses the risk of side effects, in particular, the development of refeeding syndrome. If not managed accordingly, it leads to a significant rise in morbidity and mortality. However, despite its importance, evidence-based recommendations on the management of refeeding syndrome are largely lacking, and only a few randomized controlled trials have been conducted. In light of this, the aim of this review is to raise awareness of refeeding syndrome in chronically ill patients by critically reviewing recent literature and providing a short overview as well as diagnosis and treatment algorithms of this underreported metabolic condition. In summary, recent findings suggest undergoing risk assessment and stratification for every patient receiving nutritional therapy. According to this, adaptation of energy and fluid support during the replenishment phase should be implemented in the nutritional therapy for patients at high risk. Additionally, continuous monitoring should take place, and appropriate actions should be initiated when necessary.

## 1. Introduction

Malnutrition is frequent in chronically ill medical inpatients and has been associated with several complications, such as longer hospital stays and increased morbidity and mortality [1,2]. Chronic illness is a known risk factor for malnutrition, as it leads to reduced food intake as well as unintentional weight loss and is associated with increased inflammation-driven catabolism [3]. In combination with immobilization and a marked inflammatory as well as endocrine stress response, the impairment of the nutritional condition contributes to muscle wasting and progressive deterioration of metabolic and functional status [4]. Chronically ill patients are particularly affected; more than 30% of medical patients seeking hospital care are at increased nutritional risk [5], a condition that is strongly associated with higher mortality and morbidity, functional decline as well as prolonged hospital stays [2,6]. What is more, inadequate nutrition in such populations can stimulate the deterioration of the clinical state. In fact, a single high-fat meal has proven to induce endothelial activation and dysfunction in both normal subjects and in patients with type 2 diabetes, thus further increasing both inflammation, especially through TNF-α activation, and cardiovascular risk [7]. For those patients, it is important to screen for malnutrition periodically already in the outpatient setting and especially upon hospital admission. Major international societies recommend screening as soon as possible within the first 24–48 h after admission [8]. It is very important to identify the malnourished patients or the patients at increased nutritional risk early in order to provide adequate nutritional intervention as soon as possible. Adapting nutritional therapy to individual patient’s needs improves clinical outcomes and is considered an essential part of the multimodal treatment of chronic illness [9]. Rising awareness of the benefits of a tailored nutritional intervention leads to its increasing prescription—though often without sufficient knowledge about possible adverse events [10]. As with all therapeutic interventions, inadequate nutritional therapy in a catabolic, malnourished, chronically ill patient carries risks, in particular the risk of refeeding syndrome (RFS).

The RFS is a life-threatening metabolic complication following nutrition in severely malnourished, catabolic patients, occurring more frequently in chronically ill patients [11]. It is characterized by electrolyte disturbances and fluid imbalance with consecutive sodium retention and organ dysfunction. Various vitamin deficiencies (e.g., thiamine (vitamin B1)) can contribute to the clinical picture [12]. Depending on the studied population, the incidence of RFS is reported to be as high as 15–30% [13,14,15]. However, as a universal definition of RFS is still lacking, its true incidence is unknown. In clinical practice, RFS is probably underreported due to a lack of knowledge about this condition. In a recent study among 281 physicians, only 14% were able to diagnose RFS correctly [16]. By raising awareness of this possible complication of nutritional therapy, physicians will be able to prescribe nutritional therapy, especially in the chronically ill and more vulnerable patient population, more wisely. Management of RFS is further complicated as there is a lack of robust evidence on optimal prevention and treatment strategies. Chronically ill patients are exposed to a higher risk of disability and hospital admissions and are especially vulnerable to disease-related malnutrition as well as catabolism [17]. Therefore, they deserve greater attention in the implementation of nutritional and fluid therapy, especially during the replenishment phase. In this review, we provide a summary of evidence and clinical guidance on the prevention and management of RFS for the daily clinical practice in treating chronic medical patients and managing the consequences of treatment.

## 2. Methodology

This is a narrative review. That being said, we base our recommendations on the systematic review by Friedli et al. [18] and the consensus statement of an international expert group, which was supported by evidence [19], and we include recently published literature. The systematic review of Friedli et al. was conducted in order to find evidence-based criteria regarding definition, incidence rate, risk factors, adverse events, therapy and preventive measurements of RFS. It included 45 mainly observational and also a few randomized trials with a study population of anorexic and non-anorexic patients [18]. In the next step, a group of internationally recognized nutrition specialists developed an algorithm for the management of patients with nutritional therapy in order to prevent and treat RFS, using the findings from the systematic review [19].

## 3. Pathophysiology

**Figure 1:**
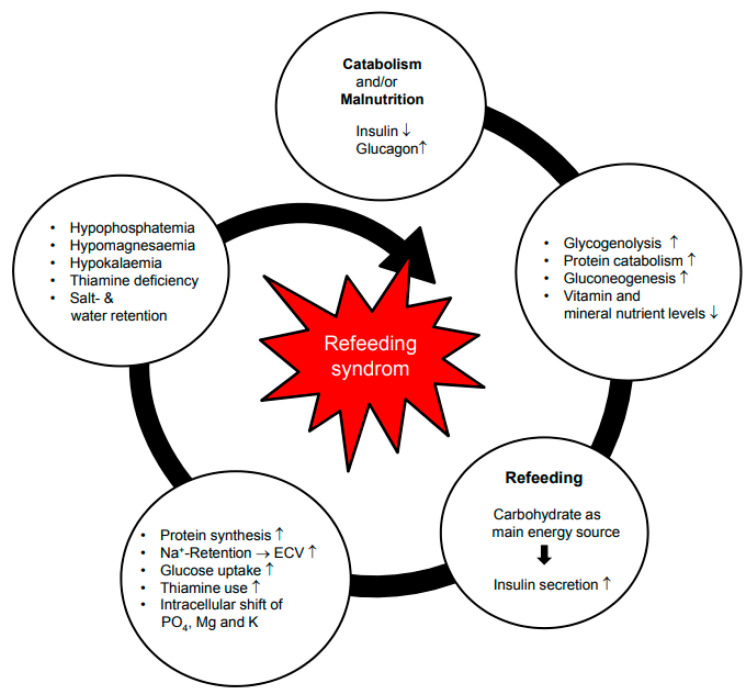
Pathophysiology of refeeding syndrome. ↑ = increased. ↓ = reduced. Bold arrow = “leading to”. Reprint with permission from Ref. [24]. 2017, Division of Diabetes, Endocrinology, Nutritional Medicine and Metabolism, University Hospital, Bern.

The exact pathophysiological mechanisms leading to RFS have not yet been definitively clarified. However, the central aspect is the switch from a catabolic to an anabolic metabolism as a normal physiological reaction upon resumption of nutrition. Hence, RFS is a physiological response to the change in metabolic status. Catabolic processes (e.g., fasting, stress reaction, inflammation) lead to a loss of intracellular ions (K, PO_4_, Mg), sodium and micronutrients [20,21]. The metabolic changes in a starved or malnourished patient after reintroduction of nutrition are mainly due to insulin stimulation following the availability of glucose. This status of hyperglycemia and hyperinsulinemia cause intracellular shifts of glucose and electrolytes and retention of sodium as well as water (decreased renal excretion), which result in characteristic RFS features, including hypophosphatemia, hypomagnesemia, hypokalemia and overload of the extracellular volume (Figure 1). In addition, thiamine pyrophosphate, which is critical in glycolysis and in the Krebs cycle, is not stored in appreciable amounts during starvation, and any acceleration of carbohydrate metabolism may precipitate its acute deficiency. This may impair glucose metabolism by producing lactate with subsequent development of lactic acidosis. Furthermore, an increase in glucose levels in critically ill patients induces overproduction of superoxide by the mitochondrial electron transport chain [22]. Consequently, the cardiac, respiratory, hematological, hepatic and neuromuscular systems are adversely affected, which can trigger clinical complications and even death [23].

## 4. Clinical Aspects

**Figure 2:**
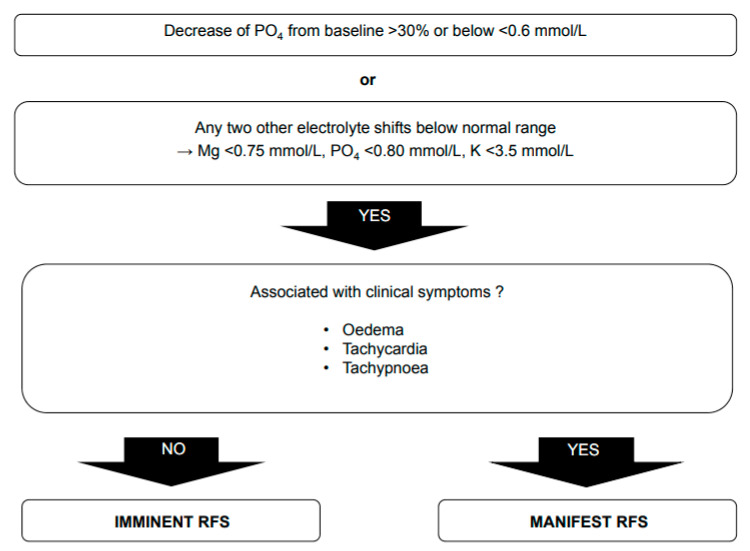
Diagnostic algorithm. Reprint with permission from Ref. [24]. 2017, Division of Diabetes, Endocrinology, Nutritional Medicine and Metabolism, University Hospital, Bern.

There is still no universally accepted definition of RFS. Its diagnosis is mainly based on plasma electrolyte disturbances in the context of nutritional replenishment during the first 72 h. Most studies consider hypophosphatemia as one of the hallmarks of RFS. As described in the pathophysiology section, starvation and the reintroduction of nutritional intake not only lead to a change in phosphate levels but also alter the levels of various intracellular ions. Therefore, the definition of RFS should not be based only on changes in phosphate levels but should also include decreases in other electrolytes. Additionally, the “ASPEN Consensus Recommendations for Refeeding Syndrome” published in 2020 defines RFS as a drop in one or any combination of electrolytes (phosphate, potassium, magnesium) or the manifestation of thiamine deficiency associated with clinical symptoms [25]. Nevertheless, it is unclear whether there is a difference between RFS and refeeding hypophosphatemia. As mentioned before, intracellular ion stores are depleted in exchange for sodium during catabolism. This reverts after the resumption of food intake. Thus, lower plasma electrolytes in acutely ill patients might actually be a consequence of lower dietary intake of these ions rather than RFS. Consequently, a differentiation between imminent RFS, when severe electrolyte derangement occurs, and manifest RFS, when a patient also presents clinical symptoms, was recently proposed [19] (Figure 2). The above-mentioned metabolic changes can lead to various clinical symptoms, which are mostly nonspecific. In daily practice, the main symptoms related to a manifest RFS are tachycardia, tachypnea and peripheral edema after exclusion of pulmonary embolism [12,23]. Other symptoms, such as impaired neuromuscular function, cardiac arrhythmia, congestive cardiac failure, encephalopathy, neuropathy and variable gastrointestinal symptoms, are possible [12,23].

## 5. State of Evidence

The association between RFS and adverse events in medical inpatients was recently shown by a secondary analysis of the EFFORT trial, which found a significant correlation between long-term mortality and other adverse clinical outcomes such as the increased risk for ICU admission and longer hospital stay [10]. In contrast, another retrospective cohort study found no correlation between RFS and increased mortality, although this study examined only short-term mortality of 30 days, and the definition criteria for RFS were based on the ASPEN guidelines, with 90% of all patients studied developing RFS [25,26].

Despite proven relevance, the state of evidence for managing RFS is largely based on observational and retrospective studies, and only a small amount of randomized controlled trials evaluating the prevention and management of RFS has been conducted. One of the only multi-center randomized control trials carried out by Doig et al. exhibited the superiority of restricted caloric feeding when managing the RFS in critically ill adult patients [27]. In this trial, 339 patients who developed hypophosphatemia (defined as serum phosphorus levels < 0.65 mmol/L) within 72 h after initiation of nutrition support were evaluated. Caloric restriction during treatment for refeeding syndrome resulted in significant improvements in overall survival time and mortality at day 60 follow-up. Moreover, a significant reduction in the incidence of major infections, airway and lung infections was observed in the reduced caloric intake group with no identified safety issues. These findings were confirmed in a subsequent retrospective study of 337 critically ill patients. The low-calorie group (which received < 50% of their goal energy for the first 3 days of refeeding with an increase in 25% of energy target per day after) had improved overall survival at day 180 and a trend toward the reduced length of stay compared to the control group (which received > 50% of their calorie goal) [28]. Additionally, in a prospective cohort study, Rio et al. showed that starvation and baseline low-serum magnesium (<0.7 mmol·L^−1^) concentration are independent predictors for the onset of RFS. Starvation, defined as a reduced nutritional intake >10 day or a weight loss >15% of body weight, was the most reliable indicator [29]. In the recent systematic review by Olsen et al. in a population aged ≥65 years, the authors showed an increased incidence of hypophosphatemia with 25% in older malnourished patients and a trend to more deaths in those patients with a higher caloric intake in the replenishment phase [30].

Preventive as well as therapeutic approaches for RFS were evaluated in the recently conducted systematic review of Friedli et al. [18]. A small group of studies stated the benefit of cautious caloric feeding as well as substitution of electrolytes in the prevention of the RFS. In addition, reduction of risk of the RFS was associated with close monitoring of the serum electrolytes. However, of the 45 studies included in the review, only a few outlined therapeutic approaches. Moreover, the majority of studies were observational, which accentuates the overall lacking state of the evidence. This lack of evidence was confirmed by another systematic review and meta-analysis published in 2021. The authors found a similarly large variability of the incidence for RFS (0–62%) as Friedli et al. Yet again, the incidence is highly dependent on the definitional criteria. Furthermore, no change in incidence was found after the definition criteria were adjusted to ASPEN (a drop of at least 10% of any electrolyte level), and no difference in incidence was found between age groups, concluding that the underlying disease is probably the most important criterion for incidence and risk of developing RFS [31]. Based on the systematic review of Friedli et al., a consensus statement was published that provided guidelines for risk stratification, management and prevention of refeeding syndrome [19].

## 6. Risk Stratification

**Figure 3:**
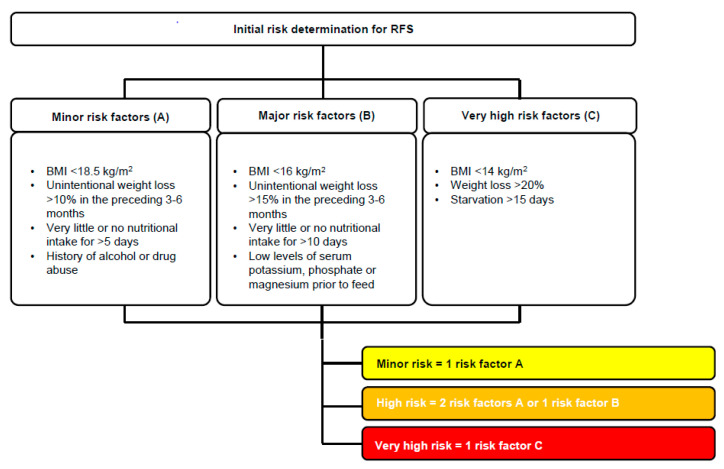
Risk stratification of refeeding syndrome. Reprint with permission from Ref. [24]. 2017, Division of Diabetes, Endocrinology, Nutritional Medicine and Metabolism, University Hospital, Bern.

Since the onset of the RFS can be very rapid (within hours after starting refeeding), the first step in the prevention of RFS is to anticipate it. That is why general recommendations emphasize awareness of patients at risk according to the National Institute for Health and Care Excellence (NICE) guidelines [11]. Identified risk factors of RFS have been summarized in the aforementioned systematic review. They include the severity of malnutrition, overaggressive beginning of nutritional support without adequate supplementation of electrolytes and thiamine and associated conditions that exacerbate electrolyte and micronutrient deficiencies, such as chronic alcoholism, gastrointestinal disorders, and poor or eccentric diets [18,23]. Certain patient groups, such as anorectic patients or hunger strike patients, are accordingly at higher risk of developing RFS in advance [18]. Regarding these factors, the consensus statement proposes risk stratification to low, high or very high risk for developing RFS [19] (Figure 3).

## 7. Management

**Figure 4:**
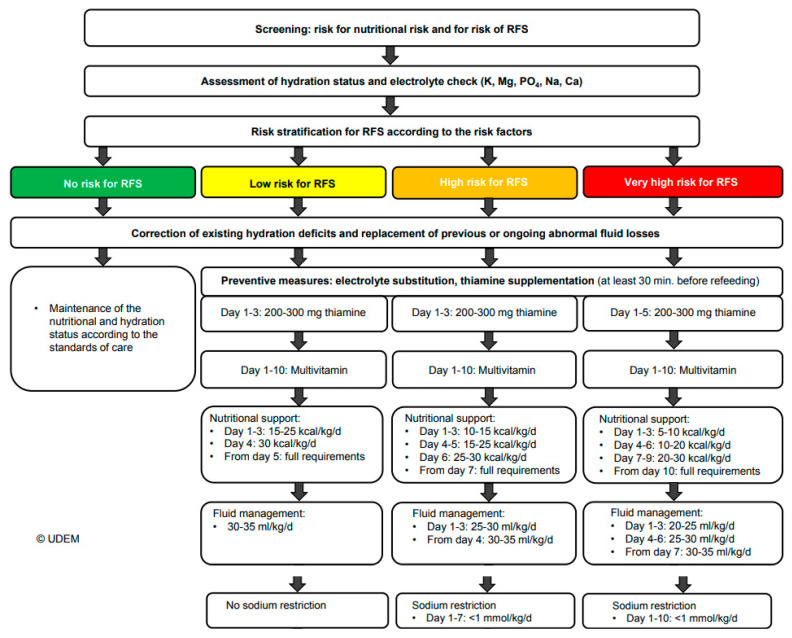
Screening and prevention algorithm. Adapted with permission from Ref. [24]. 2017, Division of Diabetes, Endocrinology, Nutritional Medicine and Metabolism, University Hospital, Bern.

Based on clinical experience as well as the summarized evidence in the literature, a standardized algorithm for managing and preventing RFS has been proposed by a group of experts [32]. Overall, in patients at risk for RFS, nutritional therapy should be started with a restrictive energy supply and then increased over the course of 5–10 days based on the previously stated risk category. Hydration deficiencies and abnormal losses (e.g., fever, vomiting, diarrhea) should be addressed at the start of a replenishment phase, and prophylactic electrolyte and vitamin substitution should be considered. Daily monitoring of serum electrolytes during the first 72 h of refeeding is also encouraged. Electrolyte substitution is advised if serum levels are lower than normal (Mg < 0.70–0.75 mmol/L, PO4 < 0.80 mmol/L, K < 3.5 mmol/L) with adaptation of daily dose according to serum values: 1–1.5 mmol/kg/d potassium, 0.2–0.4 mmol/kg/d magnesium, 0.3–0.6 mmol/kg/d phosphate. It is recommended to administer thiamine on days 1 and 3–5, multivitamins during days 1–10 and to replace specific deficiencies of trace elements. In addition, it is important to note that iron should not be given during the first 7 days of refeeding, even in the case of manifest iron deficiency due to its effect of exacerbating hypokalemia and hypophosphatemia [33]. The detailed algorithm for the assessment and prevention of RFS is stated below (Figure 4).

If RFS develops (either imminent or manifest), the substitution of the corresponding electrolytes and micronutrients is suggested. If patients suffer from manifest RFS with edema, lung failure or heart failure, an adaptation of energy and fluid intake as in high-risk patients is recommended, and an adequate treatment for these conditions should be commenced [19].

## 8. Monitoring

**Figure 5:**
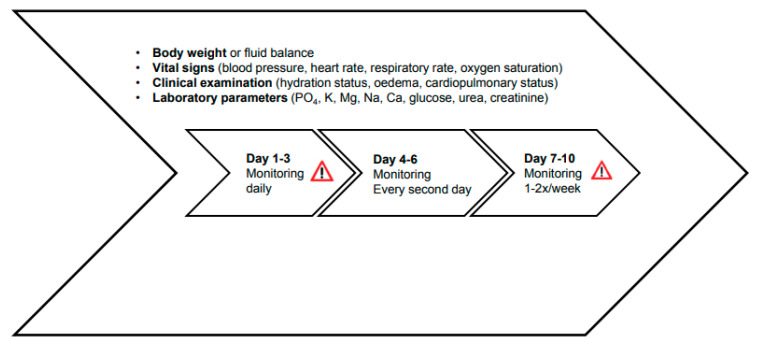
Monitoring of RFS. Reprint with permission from Ref. [24]. 2017, Division of Diabetes, Endocrinology, Nutritional Medicine and Metabolism, University Hospital, Bern.

Since the first 10 days of refeeding pose the highest risk of RFS, intensive clinical monitoring during this period is recommended. Vital signs, hydration status and analysis of laboratory parameters are essential to detect early signs of RFS such as fluid overload and organ failure. It is advised to evaluate body weight, hydration and clinical status as well as laboratory parameters daily on days 1–3 since an increase of 0.3–0.5 kg/day may be an initial sign of pathological fluid retention [34]. On days 4–6, monitoring may be conducted every second day and on days 7–10, 1–2×/week (Figure 5). Electrocardiogram during the first three days for patients at very high risk or for the ones with prior severe electrolyte imbalances (K < 2.5 mmol/L, PO4 < 0.32 mmol/L, Mg < 0.5 mmol/L) is recommended, as they may exhibit severe arrhythmia and QT-prolongation, up to Torsades de Pointes [11].

## 9. Discussion

Recognition of RFS can be particularly challenging not only due to reported unawareness of physicians but also because clinical symptoms are rather unspecific, and it is unclear in which cases electrolyte shifts and vitamin deficiency cause clinical manifestations. In addition, electrolyte imbalance is particularly common in polymorbid and chronically ill patients and is the result of other etiology [35].

In general, the literature is inconclusive, and currently, there are no universal guidelines for how to advance nutrition therapy in a safe manner. Cautious refeeding protocols may not apply to special populations, such as those with renal impairment, are meant as general guidelines and must yet be tested in randomized studies. Nevertheless, the above-proposed algorithm has already been implemented in clinical practice and could demonstrate a positive impact on the population of anorexia nervosa patients, among others. Retrospective observational analysis of 65 inpatients during a 5-year period revealed that none of the sampled patients developed RFS when the mentioned guidelines were implemented [36]. These findings support the statement that evidence-based refeeding regimens can reduce complications and prevent mortality in high-risk populations.

## 10. Conclusions

RFS is a serious life-endangering metabolic complication arising from rapid nutritional support in the replenishment phase in malnourished, catabolic patients. If not correctly diagnosed or if appropriate measures are not being taken, RFS can lead to increased morbidity and mortality. In addition, patients with chronic disease are more exposed to this complication due to catabolic metabolism and reduced nutritional intake. That is why special attention should be devoted to preventing and managing RFS in this vulnerable population. Nonetheless, the inconclusive evidence poses a challenge to developing universal, evidence-based guidelines for the diagnosis, prevention and treatment of this disorder. As long as the evidence from randomized controlled trials is still lacking, clinical management can only be based on expert consensus statements. However, a few key aspects could be defined: Risk assessment before nutritional therapy is crucial in order to prevent RFS; patients at higher risk of RFS should be administered restricted energy and fluid intake as well as vitamin and electrolyte supplementation; monitoring and management of electrolyte levels and clinical symptoms is advised for patients at risk during the first 72 h of refeeding.

That being said, future research is needed to gain in-depth knowledge, from better definitions of RFS to the standardization of the treatment protocols.

## Data Availability

All data supporting the reported results can be found in this publication.
